# Beyond *R*_0_: heterogeneity in secondary infections and probabilistic epidemic forecasting

**DOI:** 10.1098/rsif.2020.0393

**Published:** 2020-11-04

**Authors:** Laurent Hébert-Dufresne, Benjamin M. Althouse, Samuel V. Scarpino, Antoine Allard

**Affiliations:** 1 Vermont Complex Systems Center, University of Vermont, Burlington, VT 05405, USA; 2 Department of Computer Science, University of Vermont, Burlington, VT 05405, USA; 3 Département de physique, de génie physique et d’optique, Université Laval, Québec, Canada G1V 0A6; 4 Institute for Disease Modeling, Bellevue, WA 98005, USA; 5 Information School, University of Washington, Seattle, WA 98195-2840, USA; 6 Department of Biology, New Mexico State University, Las Cruces, NM 88003, USA; 7 Network Science Institute, Northeastern University, Boston, MA 02115, USA; 8 Department of Marine and Environmental Sciences, Northeastern University, Boston, MA 02115, USA; 9 Department of Physics, Northeastern University, Boston, MA 02115, USA; 10 Department of Health Sciences, Northeastern University, Boston, MA 02115, USA; 11 ISI Foundation, Turin 10126, Italy; 12 Santa Fe Institute, Santa Fe, NM 87501, USA; 13 Centre interdisciplinaire en modélisation mathématique, Université Laval, Québec, Canada G1V 0A6

**Keywords:** epidemiology, complex networks, branching processes

## Abstract

The basic reproductive number, *R*_0_, is one of the most common and most commonly misapplied numbers in public health. Often used to compare outbreaks and forecast pandemic risk, this single number belies the complexity that different epidemics can exhibit, even when they have the same *R*_0_. Here, we reformulate and extend a classic result from random network theory to forecast the size of an epidemic using estimates of the distribution of secondary infections, leveraging both its average *R*_0_ and the underlying heterogeneity. Importantly, epidemics with lower *R*_0_ can be larger if they spread more homogeneously (and are therefore more robust to stochastic fluctuations). We illustrate the potential of this approach using different real epidemics with known estimates for *R*_0_, heterogeneity and epidemic size in the absence of significant intervention. Further, we discuss the different ways in which this framework can be implemented in the data-scarce reality of emerging pathogens. Lastly, we demonstrate that without data on the heterogeneity in secondary infections for emerging infectious diseases like COVID-19 the uncertainty in outbreak size ranges dramatically. Taken together, our work highlights the critical need for contact tracing during emerging infectious disease outbreaks and the need to look beyond *R*_0_.

## 1. Introduction

In 1918, a typical individual infected with influenza transmitted the virus to between one and two of their social contacts [1], giving a value of the basic reproductive number—*R*_0_, the expected number of secondary infections by a single infected individual introduced in a completely susceptible population—of between 1 and 2. These are similar to values of *R*_0_ for the 2014 West Africa Ebola virus outbreak, yet Ebola virus disease infected a tenth of 1% of the number of individuals believed to have been infected by the 1918 influenza virus [2,3]. The two diseases are of course vastly different in symptoms and mortality, but most models to estimate the final size of an epidemic tend to ignore these features and instead focus on the actual spread through secondary infections. Similarly, the century separating the two epidemics saw vast improvements in healthcare and public health measures, as well as changes in human behaviour, which all help explain the massive discrepancy between Ebola virus disease in 2014 and influenza in 1918 [4]. There is another critical but sometimes overlooked difference between these two diseases: heterogeneity in the number of secondary cases resulting from a single infected individual. Indeed, most individuals infected with Ebola virus gave rise to zero additional infections while a few gave rise to more than 10 [5,6]. Here, we demonstrate analytically that quantifying the variability in the number of secondary infections is critically important for quantifying the transmission risk of common and novel pathogens.

The basic reproduction number of an epidemic, *R*_0_, is the expected number of secondary cases (note, we use the word ‘case’ in a generic sense to represent any infection, even if too mild to meet the clinical case definition [7]) produced by a primary case over the course of their infectious period in a completely susceptible population [8]. It is a simple metric that is commonly used to describe and compare the transmissibilty of emerging and endemic pathogens [9]. If *R*_0_ = 2, one case turns to two, on average, and two turn to four as the epidemic grows. Conversely, the epidemic will die out if *R*_0_ < 1.

Almost 100 years ago, work from Kermack & McKendrick [10–12] first demonstrated how to estimate the final size of an epidemic, integrating over all time to ignore the dynamics and focus on the final fraction of individuals reached by the epidemic, *R*(∞). Specifically, they considered a scenario such that:

(i) the disease results in complete immunity or death,
(ii) all individuals are equally susceptible,
(iii) the disease is transmitted in a closed population,
(iv) contacts occur according to the law of mass action, and
(v) the population is large enough to justify a deterministic analysis.

Under these assumptions, Kermack and McKendrick showed that an epidemic with a given *R*_0_ will infect a fixed fraction *R*(∞) of the susceptible population by solving

1.1 $$R(\infty )=-\frac{1}{R_{0}}\ln[1-R(\infty )].$$

This solution describes a final outbreak size equal to 0 when *R*_0_ ≤ 1 and increasing roughly as 1 − exp(−*R*_0_) when *R*_0_ > 1. Therefore, a larger *R*_0_ leads to a larger outbreak, which infects the entire population in the limit *R*_0_ → ∞. This direct relationship between *R*_0_ and the final epidemic size is at the core of the conventional wisdom that a larger *R*_0_ will cause a larger outbreak. Unfortunately, the equation relating *R*_0_ to final outbreak size from Kermack and McKendrick is only valid when all the above assumptions hold, which is rare in practice.

As a result, relying on *R*_0_ alone is often misleading when comparing different pathogens or outbreaks of the same pathogen in different settings [13–15]. This is especially critical considering that many outbreaks are not shaped by the ‘average’ individuals but rather by a minority of super-spreading events [13,16,17]. To more fully quantify how heterogeneity in the number of secondary infections affects outbreak size, we turn towards network epidemiology and derive an equation for the total number of infected individuals using all moments of the distribution of secondary infections.

## 2. Random network analysis

Random network theory allows us to relax some of assumptions made by Kermack and McKendrick, mainly to account for heterogeneity and stochasticity in the number of secondary infections caused by a given individual. We first follow the analysis of [18] and define

2.1 $$G_{0}(x)=\sum_{k=0}^{\infty }p_{k}x^{k}$$

as the probability generating function (PGF) of the distribution $\left\{p_{k}\right\}$ of the number of contacts individuals have (their *degree*). In other words, a randomly chosen node has a degree equal to *k* with probability *p*_*k*_. If we instead select an edge at random, the degree of the node at either of its two ends will be distributed according to *k p*_*k*_/〈*k*〉 since an edge is *k* times more likely to reach a node of degree *k* than a node of degree 1. Here $\langle k\rangle =\sum_{k=0}^{\infty }kp_{k}$ is the average degree and acts as a normalization constant. We define the *excess* degree as the number of *other* edges a node has when it has been reached via one of its edges. Since the excess degree equals the degree of a node at the end of an edge minus 1, the excess degree distribution is generated by

2.2 $$G_{1}(x)=\frac{1}{\left\langle k\right\rangle }\sum_{k=1}^{\infty }kp_{k}x^{k-1}=\frac{G_{0}^{\prime }(x)}{G_{0}^{\prime }(1)},$$

where $G_{0}^{\prime }(x)$ denotes the derivative of *G*_0_(*x*) with respect to *x*.

We now assume that the network in question is the network of all edges that *will* transmit a disease if either of the two nodes at its ends were infected. Consequently, *G*_1_(*x*) generates the number of secondary infections that individual nodes would cause if infected. Consequently, the connected component to which a node belongs (the maximal subset of nodes between which paths exist between all pairs of nodes) will be infected should that node be the first infected individual (the patient zero). In this framework, the size of the largest possible epidemic corresponds to the size of the giant connected component (GCC).

To calculate the size of the GCC, we first look for the probability *u* that following a random edge leads to a node *not* part of the GCC. For that node to not be a part of the GCC, none of its *other* neighbours should belong to it either, which occurs with probability *u*^*k*−1^ if that node has a degree equal to *k*. Since *u* is defined for any edge, we take the average over the excess degree distribution, which yields the self-consistent equation whose solution is *u*

2.3 $$u=\frac{1}{\left\langle k\right\rangle }\sum_{k=1}^{\infty }kp_{k}u^{k-1}=G_{1}(u).$$

Equation (2.3) is a condition of self-consistency since both sides describe the same quantity, *u*, under two different perspectives, which allows us to solve for *u*. The left-hand side is our definition of the probability *u* that a random edge followed in one direction does not lead to an infinite component; whereas the right-hand side calculates this probability from the perspective of the excess degree of the node reached through the random edge. The size of the GCC is a fraction of the full population *N* that we will denote *R*(∞) because it corresponds to the potential, macroscopic, outbreak size. Noting that a node of degree *k* has *no* edge leading to the GCC with probability *u*^*k*^, *R*(∞) corresponds to the fraction of nodes with at least one edge leading to the GCC

2.4 $$R(\infty )=\sum_{k=0}^{\infty }p_{k}(1-u^{k})=1-G_{0}(u).$$

Data on the distribution of secondary infections inform us about *G*_1_(*x*) directly, but our choice of *G*_0_(*x*) represents our assumptions on patient zero: is the first case different from subsequent cases? If not, we could use *G*_0_(*x*) = *G*_1_(*x*) to obtain final size estimates of a branching process as described in [13] but that would ignore the fact that patient zero was not chosen by following a person-to-person transmission link, a network bias described in [19]. When assuming a relationship between *G*_0_(*x*) and *G*_1_(*x*) as in equation (2.2), *G*_0_(*x*) will still have one degree of freedom remaining, *p*_0_, which requires further assumptions to be made to set its value (which we introduce in equation (3.8)). Putting all these different assumptions under the same framework will allow us to explicitly compare them.

Regardless of the specifics of the chosen model and of its underlying assumptions, equation (2.4) provides the size of the largest possible epidemic in the limit of infinite population size. Similarly to the Kermack–McKendrick solution, this approach provides an almost exact mapping to the final size of the dynamical spreading process without describing the temporal dynamics since we are effectively integrating over time by considering only transmissions that occur and ignoring when they occur [20]. There are however methods to use a branching process perspective or extend PGFs to temporal dynamics by considering inter-generation time [21,22].

## 3. Results

The network approach naturally accounts for heterogeneity, meaning that some individuals will cause more infections than others. The network approach also accounts for stochasticity explicitly: even with *R*_0_ > 1, there is a probability 1 − *R*(∞) that patient zero lies outside of the giant outbreak and therefore only leads to a small outbreak that does not invade the population. However, the analysis in terms of PGFs is obviously more involved than simply assuming mass-action mixing and solving equation (1.1). In fact, the PGFs *G*_0_(*x*) or *G*_1_(*x*) require a full distribution of secondary cases, which will in practice involve the specification of a high-order polynomial. Previous network models [19,23] tend to specify *G*_0_(*x*) then derive *G*_1_(*x*), but our approach focuses on secondary infections and *G*_1_(*x*) to unify the network and branching process perspectives [13,24]. Doing so clarifies our assumptions and allows us to simplify further.

To further this approach, we propose reformulating the classic network model in terms of the cumulant generating function (CGF) of secondary cases. The CGF *K*(*y*) of a random variable *X* can be written as $K(y)=\sum \kappa_{n}y^{n}/n!$, where *κ*_*n*_ are the cumulants of the distribution of secondary infections. These are useful because the cumulants are easier to interpret, i.e. *κ*_1_ is simply the average number of secondary cases *R*_0_, *κ*_2_ is the variance, *κ*_3_ is related to the skewness and *κ*_4_ is related to the kurtosis of the full distribution, etc. By definition, a PGF *G*(*x*) of a random variable is linked to *K*(*y*) through *G*(*x*) = exp[*K*(ln*x*)]. Therefore, we can replace the PGF *G*_1_(*x*) for the distribution of secondary infections by a function in terms of the cumulants of that distribution.

### 3.1. Analysis of cumulants and derivation of Kermack–McKendrick

We can easily derive Kermack and McKendrick’s result from this framework since their solution assumes a well-mixed population, which corresponds to a Poisson distribution of secondary infections. We first re-write *G*_1_(*x*) in terms of the cumulants *κ*_*n*_ as

3.1 $$G_{1}(x)=\exp\left[\sum_{n=1}^{\infty }\frac{1}{n!}\kappa_{n}{\left(\ln x\right)}^{n}\right],$$

which is a particularly convenient representation for a Poisson distribution because its cumulants *κ*_*n*_ = *R*_0_ for all *n* > 0. Moreover, since *G*_0_(*x*) = *G*_1_(*x*) in the Poisson case, the final outbreak size of the Kermack–McKendrick analysis will be set by *u*_KM_ = *G*_1_(*u*_KM_), or

3.2 $$\begin{array}{rl} u_{\mathrm{KM}} & =\;\exp\left[\sum_{n=1}^{\infty }\frac{1}{n!}R_{0}{\left(\ln u_{\mathrm{KM}}\right)}^{n}\right]=\;\exp[R_{0}(u_{\mathrm{KM}}-1)]\\ & ↪R_{\mathrm{KM}}(\infty )=1-\;\exp[R_{0}(u_{\mathrm{KM}}-1)]=1-\exp[-R_{0}R_{\mathrm{KM}}(\infty )]. \end{array}$$

Taking the logarithm of the exponential term from this last equation yields equation (1.1).

The solution to *u* = *G*_1_(*u*) gives the probability that every infection caused by patient zero fails to generate an epidemic. For more general distributions, it is useful to rewrite equation (3.1) as

3.3 $$\begin{array}{rl} u & =G_{1}(u)=\exp\left[\sum_{n=1}^{\infty }\frac{1}{n!}\kappa_{n}{\left(\ln u\right)}^{n}\right]\\ & =\exp\left[R_{0}|\ln u|-\frac{1}{2}\sigma^{2}|\ln u{|}^{2}+\frac{1}{6}\kappa_{3}|\ln u{|}^{3}-\frac{1}{24}\kappa_{4}|\ln u{|}^{4}\ldots \right] \end{array}$$

to highlight its alternating nature because the logarithm of *u* is negative (*u* is a probability) such that its *n*th power is positive when *n* is even and negative when *n* is odd.

The alternating sign of contribution from high-order moments in equation (3.3) can be interpreted as follows. A disease needs a high average number of secondary infections (high *κ*_1_ = *R*_0_) to spread, but, given that average, a disease with small variance in secondary infections will spread much more reliably and be less likely to stochastically die out. Given a variance, a disease with high skewness (i.e. with positive deviation contributing to most of the variance) will be more stable than a disease with negative skewness (i.e. with most deviations being towards small secondary infections). Given a skewness, a disease will be more stable if it has frequent small positive deviations rather than infrequent large deviations—hence a smaller kurtosis—as stochastic die out could easily occur before any of those large infrequent deviations occur.

**Figure 1.**
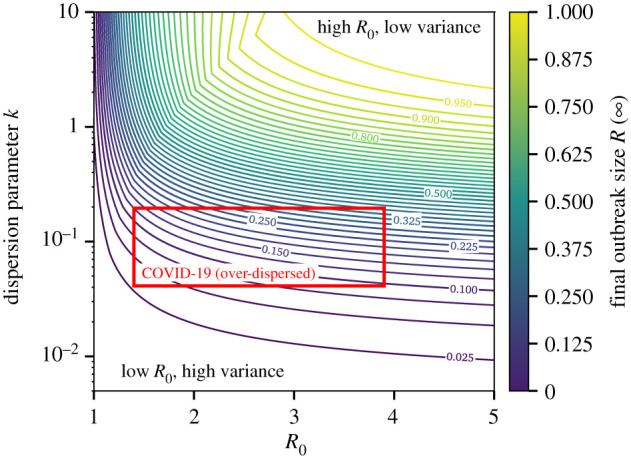
Final size of outbreaks with different average *R*_0_ and heterogeneity *k* in the distribution of secondary cases. We use a negative binomial distribution of secondary cases and scan a realistic range of parameters. The range of parameters corresponding to estimates for COVID-19 based on a binomial negative distribution in large populations is highlighted by a red box (see [25] and table 1). Most importantly, with fixed average, the dispersion parameter is inversely proportional to the variance of the underlying distribution of secondary cases. The degree of freedom, *p*_0_, is here set by setting the average number of infections around patient zero to be less than or equal to *R*_0_. The Kermack–McKendrick solution would correspond to the limit *k* → ∞, and could be more appropriate in some dense and well-mixed settings.

Our re-interpretation already highlights a striking result: higher moments of the distribution of secondary cases can lead a disease with a lower *R*_0_ to invade a population more easily and to reach a larger final outbreak size than a disease with a higher *R*_0_. This result is illustrated in figure 1.

**Table 1.** Estimates for *R*_0_ and for the negative binomial distribution dispersion parameter, *k*, used in figure 2 (^a^ and ^b^, respectively, denote 95% and 90% confidence intervals). The proportion of susceptible individuals infected as reported either in the literature or by the US Centers for Disease Control and Prevention. For severe acute respiratory syndrome (SARS) the proportion of infected was taken from serosurveys among wild animal handlers (15%) and among healthcare workers (<1%) [27]. For influenza (2009), we took data on school-aged children. For COVID-19, we present emerging evidence surrounding the final proportion of infected individuals after the first outbreak waves at the level of large communities [28,29] and a school [30], which all fall around 15%, and at the level of dense groups like a fishing vessel with a value around 86% [31]. Note that the estimates of the proportion of infected individuals, for *R*_0_ and for *k*, were not necessarily inferred from the same populations. Such information is rarely, if ever, available for the same outbreak, unfortunately. COVID-19, coronavirus disease 2019; MERS, Middle East respiratory syndrome.

disease	location	year	prop. infect.	*R*_0_	*k*	reference
MERS	global	2013	0%	0.47 (0.29–0.80)^a^	0.26 (0.09–1.24)^a^	[21,32]
SARS	global	2003	0–15%	1.63 (0.54–2.65)^b^	0.16 (0.11–0.64)^b^	[13,27,33]
smallpox	Europe	1958–1973	55%	3.19 (1.66–4.62)^b^	0.37 (0.26–0.69)^b^	[13,34]
influenza	Baltimore (USA)	1918	40%	1.77 (1.61–1.95)^a^	0.94 (0.59–1.72)^a^	[35,36]
influenza	Italy	2009	39%	1.321 (1.299–1.343)^a^	8.092 (5.170–11.794)^a^	[37,38]
COVID-19	global	2020	13–16% and 86%	2.5 (1.4–12)^a^	0.1 (0.04–1)^a^	[25,28–31,39–41]

### 3.2. Normal distributions and the impact of variance

A second useful application of the cumulants formulation involves diseases with a large reproductive number *R*_0_ whose distribution of secondary infections can be convincingly modelled by a normal distribution. Using a normal distribution for the distribution of secondary infections is only valid for very large *R*_0_ since we have to both model a discrete distribution with a continuous one and ignore negative numbers of secondary infections. The advantage of this approximation is that while the raw moments of a normal distribution are quite complicated, the cumulants are simple: *κ*_1_ is equal to the mean *R*_0_, *κ*_2_ is equal to the variance *σ*^2^ and all other cumulants are 0. We can thus write

3.4 $$G_{1}(x)=\exp\left[R_{0}\ln x+\frac{1}{2}\sigma^{2}{\left(\ln x\right)}^{2}\right]=x^{R_{0}+\frac{\sigma^{2}}{2}\ln x}$$

and solving for *u* = *G*_1_(*u*) yields

3.5 $$u=\exp\left[-\frac{2}{\sigma^{2}}(R_{0}-1)\right].$$

This equation can then be used for direct comparison of the probability of invasion of two different diseases with normal distributions of secondary infections. Given a transmission event from patient zero to a susceptible individual, disease B will be more likely to invade the population than disease A if

3.6 $$\frac{\sigma_{A}^{2}}{\sigma_{B}^{2}}<\frac{R_{0,A}-1}{R_{0,B}-1}.$$

For example, a disease with half the basic reproductive number of another will still be more likely to invade a population and lead to a larger outbreak if its variance is less than or close to half the variance of the other disease.

Altogether, the results of the previous subsections show that taking into account the contribution of these higher moments should yield different, hopefully better, estimates for the final size of real outbreaks. To test this hypothesis, we now introduce a more specific network model.

### 3.3. Negative binomial network model

We present a specific network model assuming the number of secondary infections to be distributed according to a negative binomial distribution parametrized by its average *R*_0_ and dispersion *k* [13]. Its PGF is

3.7 G1(x)=∑n=0∞(n+k−1n)[R0R0+k]n[1−R0R0+k]kxn=[1+R0k(1−x)]−k.

The general network theory formalism requires the specification of the PGF *G*_0_(*x*) that is related to *G*_1_(*x*) via equation (2.2). Specifying *G*_1_(*x*) therefore fixes *G*_0_(*x*) up to a constant and to a multiplicative factor. Without loss of generality, we set

3.8 $$G_{0}(x)=p_{0}+(1-p_{0})g_{0}(x)$$

with 0 ≤ *p*_0_ ≤ 1, *g*_0_(0) = 0 and *g*_1_(1) = 1. Equation (2.2) becomes

3.9 $$G_{1}(x)=\frac{g_{0}^{\prime }(x)}{g_{0}^{\prime }(1)},$$

from which we compute

3.10 $$g_{0}(x)=\int g_{0}^{\prime }(x)\;\text{d}x=g_{0}^{\prime }(1)\int G_{1}(x)\;\text{d}x=-\frac{kg_{0}^{\prime }(1)}{R_{0}(1-k)}{\left[1+\frac{R_{0}}{k}(1-x)\right]}^{1-k}+C,$$

with *k* ≠ 1, and where *C* and $g_{0}^{\prime }(1)$ are fixed by imposing *g*_0_(0) = 0 and *g*_1_(1) = 1. Rearranging the terms, we find that

3.11 $$g_{0}(x)=\frac{1-{\left[1-\frac{R_{0}x}{R_{0}+k}\right]}^{1-k}}{1-{\left[\frac{k}{R_{0}+k}\right]}^{1-k}},$$

from which we finally obtain

3.12 $$G_{0}(x)=p_{0}+(1-p_{0})\frac{1-{\left[1-\frac{R_{0}x}{R_{0}+k}\right]}^{1-k}}{1-{\left[\frac{k}{R_{0}+k}\right]}^{1-k}}$$

with *k* ≠ 1. The case *k* = 1 must be treated separately and yields

3.13 $$G_{0}(x)=p_{0}+(1-p_{0})\left[1-\frac{\ln[1+R_{0}(1-x)]}{\ln[1+R_{0}]}\right].$$

From equations (3.12) and (3.13), we find that the average number of secondary infections caused by patient zero is

3.14 $$G_{0}^{\prime }(1)=(1-p_{0})\frac{(1-k)R_{0}}{k}\frac{1}{{\left[\frac{k}{R_{0}+k}\right]}^{k-1}-1}$$

if *k* ≠ 1, and

3.15 $$G_{0}^{\prime }(1)=(1-p_{0})\frac{R_{0}}{\ln[1+R_{0}]}$$

if *k* = 1. The average number of secondary infections caused by patient zero can therefore be greater or smaller than *R*_0_. Since patient zero should not be expected to create *more* secondary cases than the next generation of infections, we set the value of *p*_0_ ∈ [0, 1] such that $G_{0}^{\prime }(1)$ is as close as possible to *R*_0_ whenever $G_{0}^{\prime }(1)>R_{0}$.

A large-scale epidemic is predicted by this framework [18] if

3.16 $$G_{1}^{\prime }(1)=R_{0}>1,$$

as in the analysis by Kermack & McKendrick [10–12]. Its size, *R*(∞), is computed with *G*_0_(*x*) as

3.17 $$R(\infty )=1-G_{0}(u),$$

where *u* is the solution of

3.18 $$u=G_{1}(u),$$

which we solve using the relaxation method [26] with an initial condition randomly chosen in the open interval (0, 1).

### 3.4. Comparison of estimators with empirical data

We now compare the final outbreak size estimates from equation (1.1) (Kermack and McKendrick) with estimates from equation (3.17) with a negative binomial offspring distribution (table 1). Ideally, this validation would use estimates of final outbreak size, *R*_0_ and *k* inferred from the same population, but unfortunately these are rarely, if ever, available. Similarly, once interventions are put in place and/or substantial behavioural change occurs, all methods that do not account for these effects will over-estimate the total outbreak size [42]. To attenuate some of these issues, we focus on outbreaks where no vaccine was available or before large interventions were put in place: smallpox in unvaccinated populations, the 1918 influenza pandemic, school children prior to the availability of the 2009 H1N1 vaccine, as well as for severe acute respiratory syndrome (SARS) among specific communities such as wild animal handlers (other smaller estimates correspond to healthcare workers). Importantly, focusing on smaller local outbreaks also allows us to mitigate any effect of reseeding in the same population as our approach describes a single transmission chain.

**Figure 2.**
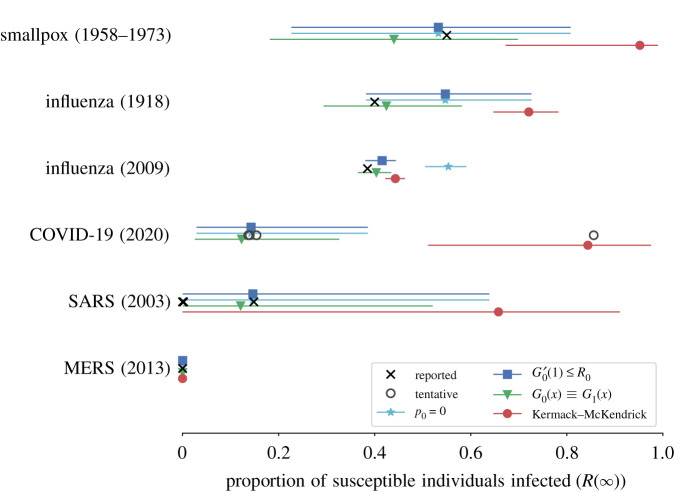
Using published estimates of *R*_0_ and the dispersion parameter *k*, we estimated the total outbreak size for six different diseases using three versions of the network approach and compared them with the classic Kermack–McKendrick solution. The confidence intervals span the range of uncertainty reported for *R*_0_ and *k*. The black markers show reported total outbreak sizes (total proportion of susceptible individuals infected) for each disease. For influenza, we report the estimated proportion of school-aged children infected. For COVID-19, we use tentative markers showing the range of attack rates measured in different contexts as there is currently no consensus for what constitutes a typical COVID-19 outbreak. We highlight though the differences between the final size estimates for COVID-19: most typify the observed over-dispersed nature of transmission, except for the outbreak on a fishing vessel (right side point) where contacts are more well mixed and thus better characterized by a Kermack–McKendrick transmission process. The red circles are the estimated proportion infected using the method developed by Kermack and McKendrick, i.e. equation (1.1). The other markers show the estimated proportion infected obtained with equation (3.17) under different assumptions about patient zero: the model described in the main text, which ensures that the expected number of secondary infections caused by patient zero is at most *R*_0_ (blue squares); the same model but assuming *p*_0_ = 0 such that no individuals have exactly zero contact (cyan stars); and a network version of [13], where *G*_0_(*x*) ≡ *G*_1_(*x*) such that patient zero is no different from subsequent patients (green triangles). See table 1 for data and additional information.

As predicted, figure 2 shows that the Kermack and McKendrick formulation consistently and significantly over-predicts the outbreak size across six different pathogens where we could find confidence interval estimates for *R*_0_ and for the negative binomial over-dispersion parameter (*k*). All network approaches produce estimates of the total outbreak size which are consistent with reported prevalence. Despite the inherent problems associated with such validations, network models appear to provide a much more reasoned estimate of the total risk to any given population, and predictions very close to the most recent seropositivity estimates for the COVID-19 outbreak in a German municipality [28] and in obstetric patients presenting for delivery [29].

## 4. Discussion

From re-emerging pathogens like yellow fever and measles to emerging threats like Middle East respiratory syndrome coronavirus and Ebola, the World Health Organization monitored 119 different infectious disease outbreaks in 2019 alone [43]. For each of these outbreaks, predicting both the epidemic potential and the most likely number of cases is critically important for efficient and effective responses. This need for rapid situational awareness is why *R*_0_ is so widely used in public health. However, our main analysis shows that not only is *R*_0_ insufficient in fully determining the final size of an outbreak, but having a larger outbreak with a lower *R*_0_ is relatively easy considering the randomness associated with most transmission events and the heterogeneity of physical contacts. To address the need for rapid quantification of risk, while acknowledging the shortcomings of *R*_0_, we use network science methods to derive both the probability of an epidemic and its final size.

These results are not without important caveats. Specifically, we must remember that distributions of secondary cases, just like *R*_0_ itself, are just as much a product of a pathogen as of the population in which it spreads. For example, aspects of the social contact network [44], metapopulation structure [45], human mobility [46], adaptive behaviour [47] and even other pathogens [48,49] all interact to cause complex patterns of disease emergence, spread and persistence. Therefore, great care must be taken when using any of these tools to compare outbreaks or to inform current events with past data. In addition, it remains a challenge to determine the final outbreak size in the absence of interventions, re-seeding, etc., and after properly accounting for the initial number of infectious individuals and the proportion of the population that is susceptible to infection. For these reasons, we focused on empirical studies that included data on the initial conditions in the population.

Figure 2 only used a few known outbreaks to validate the different approaches because data on secondary cases are rare. In practice, three types of data could potentially be used in real time to improve predictions by considering secondary case heterogeneity. First, contact tracing data, whose objective is to identify people who may have come into contact with an infectious individual. While mostly a preventive measure to identify cases before complications, it directly informs us about potential secondary cases caused by a single individual, and therefore provides us with an estimate for *G*_1_(*x*). Both for generating accurate predictions of epidemic risk and controlling the outbreak, it is vital to begin contact tracing before numerous transmission chains become widely distributed across space [50,51].

Second, viral genome sequences provide information on both the timing of the outbreak [52] and the structure of secondary cases [53]. For example, methods exist to reconstruct transmission trees for sampled sequences using simple mutational models to construct a likelihood for a specific transmission tree [54,55] and translate coalescent rates into key epidemiological parameters [56,57]. Despite the potential for genome sequencing to revolutionize outbreak response, the global public health community often struggled to coordinate data sharing across international borders, between academic researchers and with private companies [58–60]. However, the current COVID-19 pandemic has stimulated prompt and widespread sharing of genomic data; this will hopefully become standard in the future.

Third, early incidence data can be leveraged to infer parameters of the secondary case distribution through comparison with simulations. Comparing the output of agent-based simulations with reported incidence can be used to effectively sample a joint posterior distribution over *R*_0_ and dispersion parameter *k*. This approach was used by most studies referenced in table 1. Most importantly, these simulations need not be run over long periods of time to predict final outbreak size. Instead, they only need to be run over enough early data to infer the parameter estimates that are then fed into our network model to compute the final outbreak size.

As for COVID-19, figure 1 shows how the width of the confidence interval on our prediction for the final outbreak size mostly stems from uncertainty in the heterogeneity of secondary infections, i.e. the dispersion parameter *k*. Note that the estimates for *R*_0_ and *k* used here are from population-level estimates (table 1) and are therefore not representative of COVID-19 in all contexts. With limited heterogeneity, our predictions would have been closer to classic mass-action forecasts and the current pandemic of COVID-19 would probably have been a consequence not only of *R*_0_ but also of the homogeneity of secondary infections: each new case steadily leading to additional infections. However, we note that emerging evidence, taken from a serosurvey in the municipality of Gangelt, Germany [28], and from universal testing in all obstetric patients presenting for delivery at two hospitals [29], suggests that the final size for a single, established COVID-19 transmission chain is around 15% of the population, which is both in agreement with estimates from our approach and far below the final size predicted by the Kermack and McKendrick formulation. With recent large estimates for its heterogeneity, the observed transmission could be mostly maintained by so-called ‘super-spreading events’, which could be easier to manage with contact tracing, screening and infection control [61,62].

In conclusion, we reiterate that, when accounting for the full distribution of secondary cases caused by an infected individual, there is no direct relationship between *R*_0_ and the size of an outbreak. We also stress that both *R*_0_ and the full secondary case distribution are not properties of the disease itself, but are instead set by properties of the pathogen, the host population and the context of the outbreak. This is best exemplified by the widely different attack rates of COVID-19 observed in figure 2 between the fishing vessel (85.6%) and the school (13.7%). Both populations were roughly of the same size but contacts in the former are denser and much more homogeneously mixed, leading to an outbreak consistent with the Kermack–McKendrick solution while contacts in the latter follow heterogeneous classroom and age patterns leading to a lower outbreak size. Our methodology can straightforwardly translate any of these estimates of transmission heterogeneity into epidemic forecasts. Altogether, predicting outbreak size based on early data is an incredibly complex challenge but one that is increasingly within reach owing to new mathematical analyses and faster communication of public health data.

## Data accessibility

See https://github.com/Emergent-Epidemics/beyond_R0 for data and scripts.
